# Complement mediates neuroinflammation and cognitive decline at extended chronic time points after traumatic brain injury

**DOI:** 10.1186/s40478-021-01179-6

**Published:** 2021-04-20

**Authors:** Khalil Mallah, Christine Couch, Mohammed Alshareef, Davis Borucki, Xiaofeng Yang, Ali Alawieh, Stephen Tomlinson

**Affiliations:** 1 Department of Microbiology and Immunology, Medical University of South Carolina, 173 Ashley Avenue, BSB 204, MSC 504, Charleston, SC 29425 USA; 2 Department of Health Sciences and Research, College of Health Professions, Medical University of South Carolina, Charleston, SC 29425 USA; 3 Department of Neurological Surgery, Medical University of South Carolina, Charleston, SC 29425 USA; 4 Department of Neurosciences, Medical University of South Carolina, Charleston, SC 29425 USA; 5 Medical Scientist Training Program, Medical University of South Carolina, Charleston, SC 29425 USA; 6 Department of Neurosurgery, Emory University School of Medicine, Atlanta, GA 30322 USA; 7 Ralph Johnson VA Medical Center, Charleston, SC 29401 USA

**Keywords:** Traumatic brain injury, Complement inhibition, Cognitive decline

## Abstract

Traumatic brain injury (TBI) can result in progressive cognitive decline occurring for years after the initial insult, and for which there is currently no pharmacological treatment. An ongoing chronic inflammatory response after TBI is thought to be an important factor in driving this cognitive decline. Here, we investigate the role of complement in neuroinflammation and cognitive decline for up to 6 months after murine TBI. Male C57BL/6 mice were subjected to open head injury using a controlled cortical impact device. At 2 months post TBI, mice were moved to large cages with an enriched environment to simulate rehabilitation therapy, and assigned to one of three treatment groups: 1. vehicle (PBS), 2. CR2Crry (3 doses over 1 week), 3. CR2Crry (continuous weekly dose until the end of the study). The study was terminated at 6 months post-TBI for all groups. Motor and cognitive function was analyzed, with histopathological analysis of brain tissue. Measured at 6 months after TBI, neither of the complement inhibition paradigms improved motor performance. However, mice receiving continuous CR2Crry treatment showed improved spatial learning and memory compared to both mice receiving only 3 doses and to mice receiving vehicle control. Analysis of brain sections at 6 months after injury revealed ongoing complement activation in the control group, with reduced complement activation and C3 deposition in the continuous CR2Crry treatment group. The ipsilateral hemisphere of continuously treated animals also showed a decrease in microglia/macrophage and astrocyte activation compared to vehicle. There was also increased astrocytosis in the contralateral hippocampus of vehicle treated vs. naïve mice, which was reduced in mice continuously treated with CR2Crry. This study demonstrates continued complement mediated neuroinflammation at extended chronic time points after TBI, and extends the potential treatment window for complement inhibition, which has previously been shown to improve outcomes after murine TBI.

**Supplementary Information:**

The online version contains supplementary material available at 10.1186/s40478-021-01179-6.

## Introduction

Studies have revealed a causative link between TBI and dementia, especially early-onset dementia [35]. Following TBI, the main clinical manifestations of cognitive impairment include attention deficit and memory loss [33]. Following a severe TBI, the risk of developing dementia is 2-folds higher compared to non-injured individuals [36]. Among US military veterans, even a mild TBI which was not accompanied by loss of consciousness, was associated with more than a 2-fold increase in risk for developing dementia [7].

Inflammation plays a key role in the progressive degenerative events that occur after TBI. The complement system is recognized as an important contributor to neuroinflammation and secondary injury processes after TBI through mechanisms that promote neuronal loss, edema, and inflammatory cellular infiltrate [2, 4, 12, 19, 41]. The complement system can be activated via three main activation pathways: the classical, lectin, and alternative pathway, with all pathways converging at C3 cleavage which yields C3a and C3b. The C3b product, which covalently attaches to complement activating cell membranes, is involved in further propagating complement activation, and its degradation products (such as iC3b and C3d) remain attached to cell surfaces and function as opsonins for complement receptors on immune cells. The other biologically active products of complement activation are the anaphylatoxins (C3a and C5a) and the cytolytic membrane attack complex (MAC). The role of complement in acute phases after TBI is well studied, but few studies have addressed the role of complement in the chronic phase after TBI. We recently investigated the role of complement and the effect of complement inhibition for up to 2 months after TBI. In these previous studies, we demonstrated that transient inhibition of complement at 2 months after TBI interrupted a degenerative neuroinflammatory response and reversed cognitive decline measured at 3 months after TBI [2]. However, unresolved is whether transient inhibition of complement is protective at more chronic time points after TBI, and specifically whether transient complement inhibition breaks a cycle of chronic complement activation and neuroinflammation that otherwise leads to cognitive decline. An alternative hypothesis, that is investigated here, is that complement activation signals persist, and at later time points after transient complement inhibition these signals lead to reactivation of a neuroinflammatory response and ongoing cognitive decline.

We investigated the role of complement in behavioral and pathophysiological outcomes at chronic time points after murine TBI in a clinically relevant paradigm. The complement inhibitor used in these studies is CR2Crry, a fusion protein between complement receptor 2, which specifically targets C3 opsonins deposited at sites of complement activation, and mouse Crry, which inhibits all activation pathways at the C3 activation step [4, 6, 44]. Previous studies have investigated C3 inhibitors in brain injury models, albeit in acute treatment paradigms and with follow up not extending past 7 days. Following weigh drop injury, rats treated with soluble CR1, which is the human ortholog of mouse Crry, resulted in decreased neutrophil accumulation in the brain [28]. Crry-Ig (an Fc region linked to Crry) improved neurological score after closed head injury, which correlated with up-regulation of neuroprotective genes in the injured hemisphere such as Bcl-2 and CD55 [30]. Using induced neural stem cells (iNSCs) treated with serum from TBI injured mice, Crry was shown to exert a neuroprotective effect by interaction with Akt as measured 12 hours post-trauma [21]. The same study showed that intracerebral-transplanting of pretreated iNSCs with CR2-Crry also increased the level of Crry expression in astrocytes and neurons derived from these cells and attenuated complement-mediating injury following closed head TBI. Also, CR2 deficient mice were shown have decreased levels of deposited C3 after closed head injury, accompanied with a level of protection [34]. In a study that extended post-trauma analysis out to 4 weeks, soluble Crry expression in GFAP-sCrry transgenic mice resulted in a decrease in neurological severity score and improved blood-brain barrier function [40].

In the current study, we investigate how complement activation and complement inhibition determines behavioral and pathophysiological outcomes at 6 months after murine TBI, and we investigate different paradigms of complement inhibition administered chronically after TBI.

## Methods

### Animal care and housing

Male C57BL/6 mice (Jackson Labs) underwent controlled cortical impact (CCI) at 11–12 weeks of age, and then subjected to different treatment paradigms. All animal studies were approved by the Institutional Animal Care and Use Committee (IACUC) at the Medical University of South Carolina and Ralph H. Johnson VA Medical Center. Mice were subjected to a 12:12 light: dark cycle and all behavioral testing was performed during the light cycle.

### Controlled cortical impact (CCI)

Surgeries were performed as previously described [4, 37]. In brief, coordinates for CCI were set halfway between the bregma and lambda points of the brain and 0.5 mm lateral to the midline in the right hemisphere of the brain. Following craniotomy, contusion was performed with a pneumatic impactor device (Infinite Horizon, Precision Scientific Inc.) with impactor tip size of 3mm. The impact was delivered to the brain on intact dura with parameters as follows: depth = 2.5 mm, velocity = 5.25 m/sec, dwell time of 100 ms, and with a 10° angle. An atlas image of injury location along with photographs of an injured brain at day 180 and a naïve age-matched brain is found in Additional File 1a.

### Recombinant protein production, purification, and study design

Preparation of the recombinant complement inhibitor CR2Crry has been previously described [6]. All preparations were checked for endotoxin, and complement inhibitory activity confirmed as described [6, 26]. Animals were treated via intraperitoneal (IP) injection at a dose of 10 mg/kg CR2Crry in phosphate-buffered saline (PBS), which was found optimal for the suppression of complement activity in previous acute TBI studies [4]. All treatments started 2 months after the TBI insult and at that time animals were moved from standard size cages to Enriched Environment (EE) cages in order to mimic voluntary motor and cognitive rehabilitation. These cages were double the size of normal housing cages and equipped with ladders, running wheels, plastic tubes, and wooden toys. The setup of an EE cage is shown in Additional File 1b. Groups of animals were subject to different treatment paradigms. One group received a total of 3 doses of CR2Crry every other day (transient group). Another group received continuous treatment, with animals first receiving 3 doses of CR2Crry every other day, followed by a single weekly dose until 6 months after injury (i.e. 4 months after the first administered dose). Vehicle animals received a PBS IP injection in the same schedule as continuous treatment. Each dose of CR2Crry was 10 mg/kg, in accordance with a previous study showing efficacy of a single dose at this concentration when measuring acute outcomes [4], and frequency of dosing was based on our previous dosing schedule in a 2 month paradigm [2].

### Behavioral testing

*Barnes maze:* This task was used to asses spatial learning and memory as previously described [4, 38]. For analysis, mice were recorded via video camera and data was obtained using the Noldus EthoVision XT system in which total path length was computed. This task was performed on all groups in the last 8 days of the study. *Grip Strength:* Forelimb grip strength was assessed using a grip strength meter as described [11], with each animal allowed 10 trials, with the mid 6 values of the 10 trials used to compute an average strength (force). This task was performed on vehicle and CR2Crry treated mice 180 days after TBI. *Open Field Ambulation:* Distance moved was monitored in an open field arena equipped with a Noldus EthoVision XT system that allowed for automated recording, animal tracking, and quantitating behavioral parameters such as distance traveled. Animals were placed in one of the corners of the box and allowed to move freely and explore for 15 min and distance moved was measured. Recordings were acquired 180 days after TBI for vehicle, CR2Crry treated mice, and naïve mice of corresponding age. *Gait analysis (Catwalk):* Gait function was performed using the automated CatwalkXT system (Noldus Technology Company) as described [13]. Briefly, this system consists of an enclosed walkway on an illuminated glass plate. As the mouse passes from one end of the walkway to the other, scattered light from contact between each paw and the glass is recorded by a high-speed camera set under the glass. Based on the obtained videos of light scattered within a defined area, software computes several gait parameters for each of the hind and front limbs including: print area, base of support, max contact intensity, paw swing, and others for each of three runs, with averaging to obtain values per trial. Recording area was defined with a 10 x 20 cm area and each trial consisted of 3 runs. Run capture duration had a minimum time of 0.5 sec and maximum time of 15 sec, thus any run less than 0.5 sec or greater than 15 sec was excluded. Any run with more than 60% variation was also excluded. 180 days after TBI, recordings were acquired for vehicle and CR2Crry treated mice, and the same task performed for age-matched naïve mice.

### Brain procurement and tissue preparation

Animals were euthanized by isoflurane overdose followed by intracardiac perfusion with cold PBS and subsequently with 4% paraformaldehyde in PBS. Brains were extracted and fixed overnight in 4% paraformaldehyde at 4°C. Brains were then immersed in 30% sucrose in PFA and cut into 40-µm thick coronal sections as previously described [4].

### Histological analysis

For assessment of lesion volume, 8 serial sections of 40-µm thickness and 700–800 µm apart were selected, mounted on gelatin-coated slides, and following 2 hours of air drying, stained using cresyl violet as previously performed [4, 45]. Tissue sections were imaged at a 4x magnification using Keyence BZ-X710 microscope while applying the tile scan option followed by stitching of all tiles to obtain a full scan of each brain section. To ensure an unbiased approach to quantify lesion, an investigator blinded to treatment groups mapped areas of tissue loss. Lesion volume, ipsilateral tissue remaining volume, and contralateral tissue volume were computed after obtaining area data using NIH Image J from the 8 serial sections. For analysis, percent lesion was computed by dividing the summation of lesion volume from all 8 serial sections by the summation of contralateral tissue volume from all sections multiplied by 2. 3D representative volume filled brain images were prepared from the 8 Nissl-stained sections using the “Free-D” software [5, 10].

### Immunofluorescence (IF) staining and imaging

IF staining was performed using 40-µm thick slices prepared as above, and sections chosen based on stereological coordinates lying between (+)0.5 mm and (-)2.5 mm relative to bregma. From each animal, 2 full brain slices were selected with matching coordinates [Bregma -1.46 mm (+/- 0.04) and -0.18 mm (+/-0.04)] to stain using free floating technique as previously described [2, 4]. Location of the selected brain sections is shown in a sagittal view of the mouse brain in Additional File 2. The following primary antibodies were used: anti-C3 (Abcam, Cat. #: ab11862, 1:200) (which recognizes intact C3 as well as activated cleaved products including C3b, iC3b, C3d, and C3dg), anti-Iba1 (Invitrogen, Cat. #: PA5-21274, 1:80) (for microglia/macrophage), anti-GFAP (Invitrogen, Cat. #: 13-0300, 1:200) (for astrocytes), and anti-NeuN (Abcam, Cat. #: ab104225, 1:200) (for Neurons). Secondary antibodies used in this study were: donkey anti-rat AlexaFluor 555 nm (Abcam, Cat. #: ab150154, 1:200), donkey anti-goat AlexaFluor 647 nm (Invitrogen, Cat. #: A32849, 1:200), donkey anti-rabbit AlexaFluor 488 nm (Invitrogen, Cat. #: A-21206, 1:200), donkey anti-rat AlexaFluor 488 nm (Invitrogen, Cat. #: A-21208, 1:200), and donkey anti-rabbit AlexaFluor 555 nm (Invitrogen, Cat. #: A-31572, 1:200). Epifluorescence imaging for full scans was performed using 10X magnification on the Keyence BZ-X710 microscope using the tile scan option followed by stitching of all tiles. Quantification of signal was performed using NIH ImageJ by a blinded investigator. Higher magnification images were acquired using a Zeiss LSM 880 confocal microscope using a 40X water objective to capture images at same coordinates with regard to injury site across all samples and different treatment conditions. These images were acquired using the Z-stack feature of the microscope covering 30–40 µm thick regions with a resolution of 1µm per slice within the stack. Images were processed and signal was normalized on the Zen (Zeiss) software and images were analyzed using NIH ImageJ. 3D reconstruction of the 40X imaged fields was than performed on Imaris 9.3 (Bitplane) software. The imaris scrip was used with uniform setting across all slices to obtain surfaces of reconstructed neurons, microglia, and complement C3. The spot analysis was then employed to localize complement deposition fragments and the number of interactions with other surfaces (i.e. microglia and neurons). The number of C3/neuron interactions and the C3/neuron/microglia interactions was then reported.

### Statistical analysis

Statistical analysis and data representation was achieved using the GraphPad Prism 8 (GraphPad, CA) software. Details of statistical tests used for different analyses are described in figure legends. All data in the manuscript are represented as mean ± SEM and P values < 0.05 were considered significant. Power sample size estimation was done as previously and was based on prior studies [2, 4]. This was achieved using G*Power 3.1 [18] and with an acceptable power range of 80–90%. Outliers were defined as having a value greater than the 75 per + 1.5xIQR or lower than 25 per - 1.5xIQR. 75 per corresponds to the 75th percentile of the data, 25 per corresponds to the 25^th^ percentile of the data, and IQR is the interquartile range which corresponds to the difference between the 75th and 25th percentiles.

## Results

### Complement inhibition in chronic phases post TBI improves cognitive performance and decreases lesion volume

**Fig 1.**
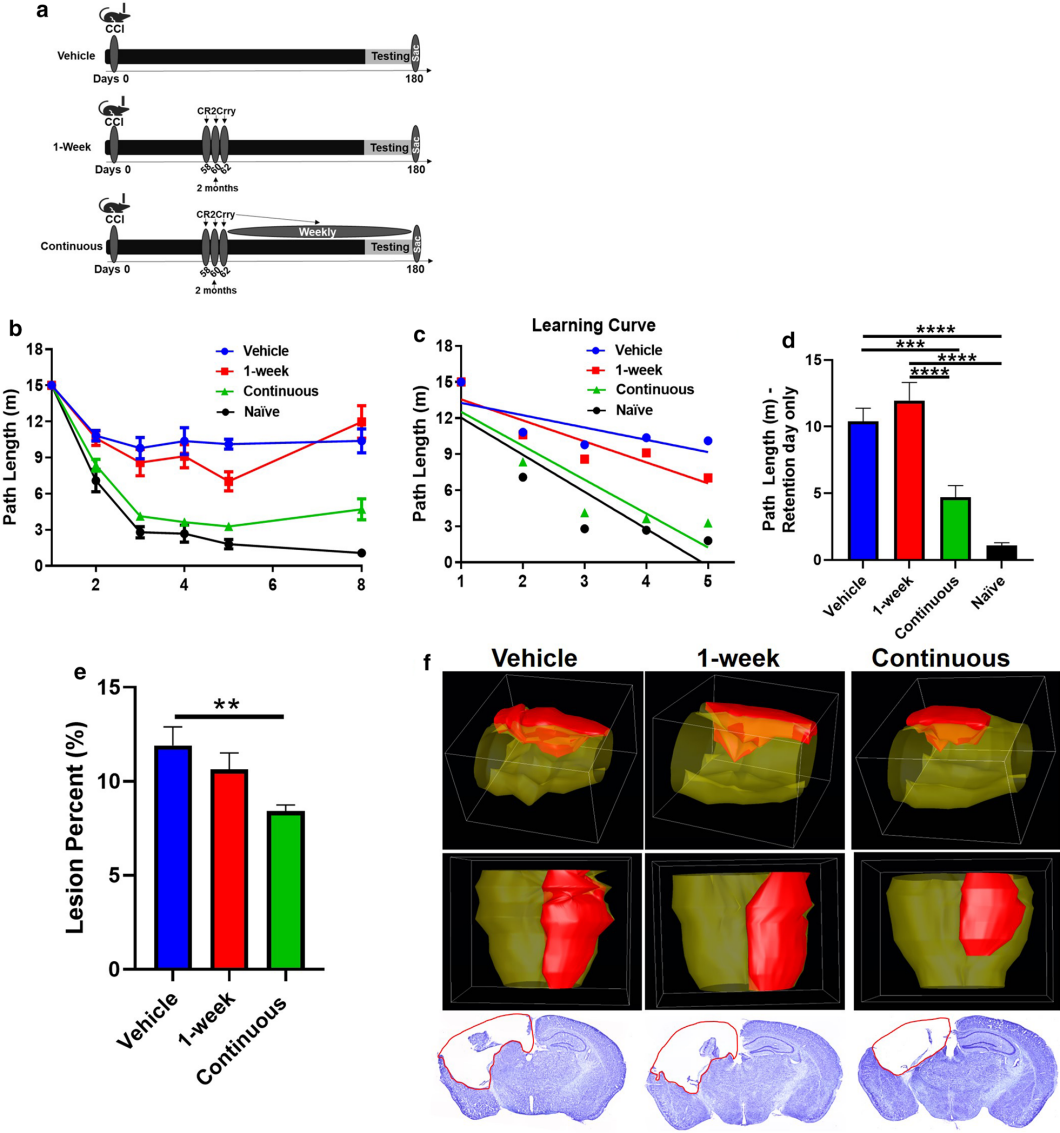
Complement inhibition in chronic phases post TBI improves cognitive performance and decreases lesion volume. **a** Workflow of different treatment paradigms used in the study. **b** Spatial learning and memory assessed using Barnes maze represented by display of path length (distance travelled in m). **c** Learning curve for the first 5 days of the task. **d** Comparison of memory retention as assessed by distance travelled represented by path length only on retention day (day 8). For retention day data, statistical analysis was carried out using one-way ANOVA with Bonferroni correction for multiple comparisons. Unpaired T-test: continuous vs. vehicle. Error bars = Mean +/- SEM. *p < 0.05, **p < 0.01, ***p < 0.001, ****p < 0.0001. Vehicle (n = 12), 1-week (n = 9), continuous (n = 12), and Naïve 9 months (n = 9). **e** Lesion volume for the different groups represented as lesion % of brain. One-way ANOVA with Bonferroni correction for multiple comparisons. Unpaired T-test: continuous vs. vehicle. Error bars = Mean +/- SEM. **p < 0.01. Vehicle (n = 10), 1-week (n = 9), and continuous (n = 12). **f** 3D reconstruction of representative brains from each group, along with sample 2D images from Nissl stains presented in the bottom row.

To investigate the dynamics of complement activation and its role in promoting chronic neuroinflammation and cognitive decline, we investigated two treatment paradigms of complement inhibition applied in the chronic phase after TBI. In one paradigm, CR2Crry was administered in 3 doses over 1-week starting at 2 months after TBI, and animals monitored until euthanized at 6 months post-TBI (termed 1-week treatment). In the second paradigm, following a 3 dose treatment with CR2Crry as above, treatment was continued on a weekly basis through 6 months after TBI (termed continuous treatment). A vehicle control (PBS) group was also included (refer to schematic, Fig. 1a). After TBI, mice were allowed to recover while being housed in an enriched environment that models cognitive and motor rehabilitation, a frequent clinical scenario (See “Methods” section, Additional File 1b).

Prior studies have shown persistent impairment in spatial learning and memory after TBI [2, 4, 16, 20, 32], and we therefore measured performance of all groups on Barnes Maze task starting at day 172 after TBI and compared performance to age-matched naïve mice (Fig. 1b–d). During the learning phase of the task (first 5 days), mice that received continuous CR2Crry treatment showed a significantly steeper learning slope over time (Path length vs. Time) compared to both vehicle and 1-week treatment groups (Fig. 1b,c, and Additional File 3). There was no difference between vehicle and 1-week treatment groups. Similarly, on retention day testing, mice treated continuously with CR2Crry showed no difference in retention of learned memory compared to naïve, and they performed significantly better than 1-week and vehicle treatment groups (Fig. 1d). Using volume reconstruction of Nissl stained brain sections, we also demonstrated that continuous, but not 1-week treatment, reduced lesion volume measured 6 months after TBI, corresponding to cognitive outcome measures (Fig. 1e,f).

To note, the rationale for using age-matched naïve animals versus sham is that skin incision and craniotomy can lead to proinflammatory, morphological, and behavioral damage compared to naïve animals, and therefore can be considered a component of brain injury [15].

### Complement inhibition in chronic phases post TBI does not improve motor performance

**Fig 2.**
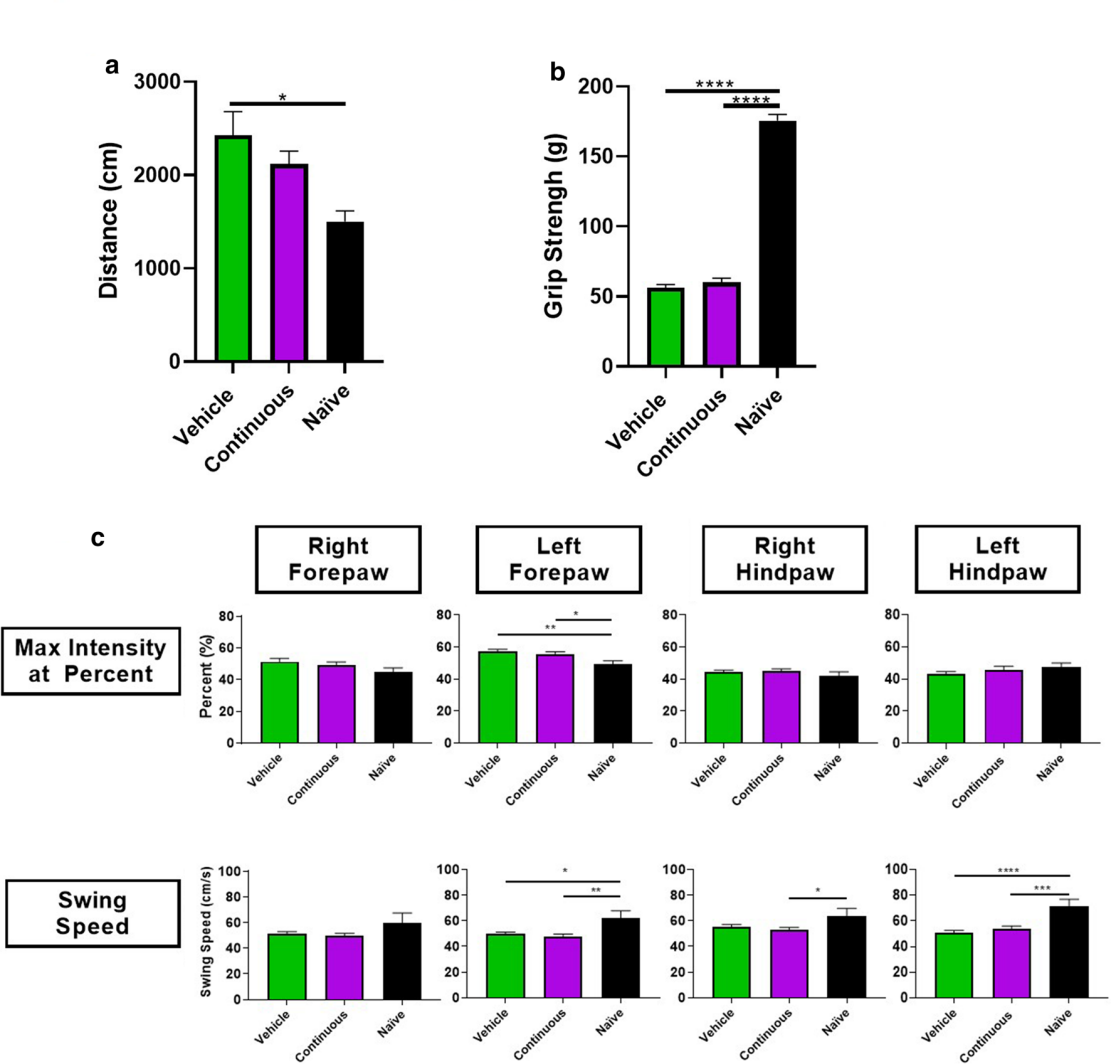
Complement inhibition in chronic phases post TBI does not improve motor performance. **a** Distance travelled (in cm) in open field ambulation test for vehicle (PBS), continuous CR2Crry treatment, and age-matched naïve WT mice (9 months of age). One-way ANOVA with Bonferroni correction for multiple comparisons. Unpaired T-test: continuous versus vehicle. Error bars = Mean +/- SEM. *p < 0.05. Vehicle and CR2crry (n = 15–16/group), naïve 9 months (n = 10 group).**b** Forearm grip strength test for vehicle, continuous CR2Crry treatment, and age-matched naïve WT mice (9 months of age). One-way ANOVA with Bonferroni correction for multiple comparisons. Unpaired T-test: continuous vs. vehicle. Error bars = Mean +/- SEM. Vehicle and CR2crry (n = 17–18/group), naïve 9 months (n = 10). **c** Catwalk parameters: Max intensity at percent and swing speed for vehicle, continuous CR2Crry treatment, and age-matched naïve WT mice (9 months of age). One-way ANOVA with Bonferroni correction for multiple comparisons. Unpaired T-test: continuous vs. vehicle. Error bars = Mean +/- SEM. *p < 0.05, **p < 0.01, ***p < 0.001, ****p < 0.0001. Vehicle/Continuous (n = 18), naïve 9 months (n = 10).

The continuous CR2Crry treatment paradigm that improved cognitive outcome and decreased lesion size was also used to evaluate motor performance after 6 months post injury (with the last 4 months of recovery being in an enriched environment). Control vehicle treatment and naïve age matched mice (9 months) were included in this study. Three behavioral tasks were used to evaluate motor function, namely open field ambulation, forearm grip strength, and gait analysis (Catwalk XT). These tasks cover different domains of motor activity that include fine motor strength, balance, coordination and speed. None of the tests showed any differences between vehicle and CR2Crry treated groups, but some tests did show persistent motor dysfunction at 6 months after TBI compared to naïve controls (Fig. 2).

### Complement deposition, microgliosis and astrocytosis are reduced with continuous complement inhibition

**Fig 3.**
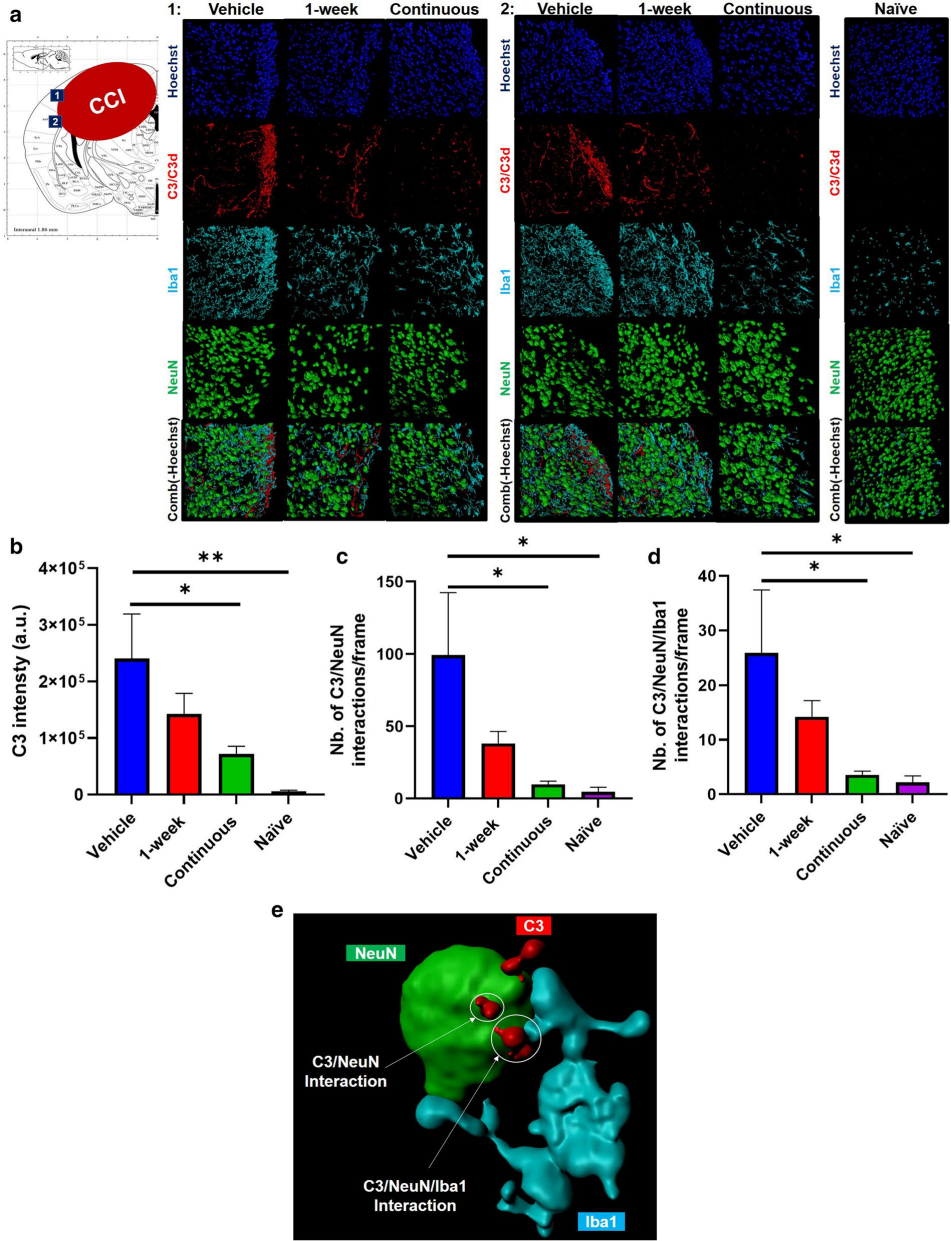
Complement C3 deposition decreases with continuous CR2Crry treatment. **a** Representative Imaris images of processed 40x z-stacked immunofluorescence images acquired by confocal microscopy from two regions adjacent to site of TBI impact (as shown in the atlas image represented in this figure) from vehicle treatment, 1-week CR2Crry treatment, continuous CR2Crry treatment, and naïve mice (9 months). Hoechst (blue), C3 (red), NeuN (green), and Iba-1 (aqua). **b** Quantification of C3 intensity. **c** Quantification of number of C3/NeuN interactions per frame. **d** Quantification of number of C3/NeuN/Iba1 interaction per frame. One-way ANOVA with Bonferroni correction for multiple comparisons. Unpaired T-test: continuous vs. vehicle. Error bars = Mean +/- SEM. *p < 0.05, **p < 0.01. n = 6–9/group. **e** 3D representative example of interactions (white arrows) between C3 (red)/ NeuN (green) and C3 (red)/ NeuN (green)/ Iba1 (aqua).

We next investigated whether the differences between cognitive outcomes in 1-week versus continuous CR2Crry treatment groups correlated with differences in a neuroinflammatory response at 6 months after TBI. We first used high resolution immunofluorescence microscopy to analyze complement (C3) deposition, together with analysis of C3/neuron and C3/neuron/microglia colocalization within the perilesional area (Fig. 3a). C3 deposition was lower in brains from mice treated continuously with CR2Crry compared to vehicle treated mice. Brains from 1-week treated mice also appeared to show decreased C3 deposition, but the difference with vehicle did not reach significance (*p* = 0.58) (Fig. 3b). Since we previously demonstrated that microglia play a role in phagocytosis and elimination of C3d tagged synapses at earlier time points after TBI [2], we also performed a colocalization analysis. Continuous CR2Crry treatment, but not 1-week treatment, showed a significant decrease in number of C3/NeuN co-localization events, as well as reduced C3/NeuN/Iba1 interactions when compared to vehicle controls (Fig. 3c, d). These data are in support of a similar mechanism of neuron/synapse elimination occurring as long as 6 months after TBI. A 3D representative image of C3/NeuN and C3/NeuN/Iba1 interactions is shown in Fig. 3e. A rotating video of the 3D structures is in Additional File 4.

**Fig 4.**
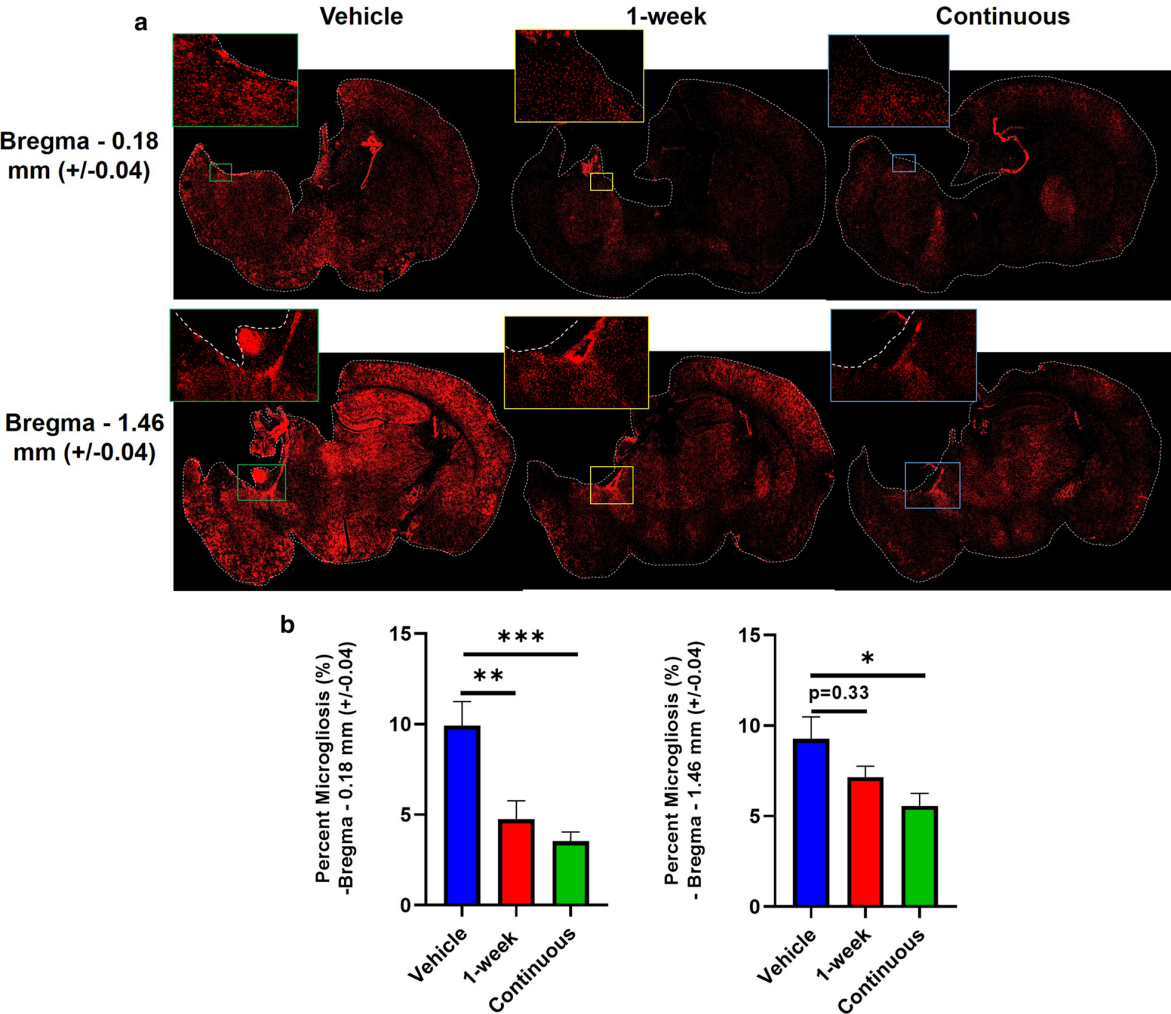
Continuous CR2Crry treatment decreases area of microgliosis in the ipsilateral hemisphere. **a** 4x stitched immunofluorescence images of Iba-1 (red) microglia/macrophage staining in the three conditions of the study: vehicle, 1-week CR2Crry, and continuous CR2Crry from two different brain coordinates: Bregma − 0.18 mm (+/-0.04) and Bregma − 1.46 mm (+/-0.04). **b** Quantification of stained images. One-way ANOVA with Bonferroni correction for multiple comparisons. Unpaired T-test: continuous vs. vehicle. Error bars = Mean +/- SEM. *p < 0.05, **p < 0.01, ***p < 0.001. n = 8–11/group.

We analyzed microgliosis at 6 months post TBI by Iba1 staining using unbiased stereology for selection of brain sections from different animals within the area of injury based on location of Bregma as mapped to the Paxino’s brain atlas. Iba1 will not distinguish between microglia and infiltrating macrophages, but the term microgliosis is used here for brevity. Slices from locations corresponding to Bregma − 1.46 mm (± 0.04) (hippocampal plane) and − 0.18 mm (± 0.04) (rostral to hippocampus) are shown (Fig. 4a). Sections more caudal to the hippocampus showed little to no evidence of microgliosis. Compared to vehicle controls, the area of microgliosis was significantly reduced in brains from mice continuously treated with CR2Crry at both stereotactic locations. Brains from 1-week treatment group showed a significant decrease in the area of microgliosis at Bregma − 0.18 mm, but not at Bregma − 1.46 mm (Fig. 4b).

**Fig 5.**
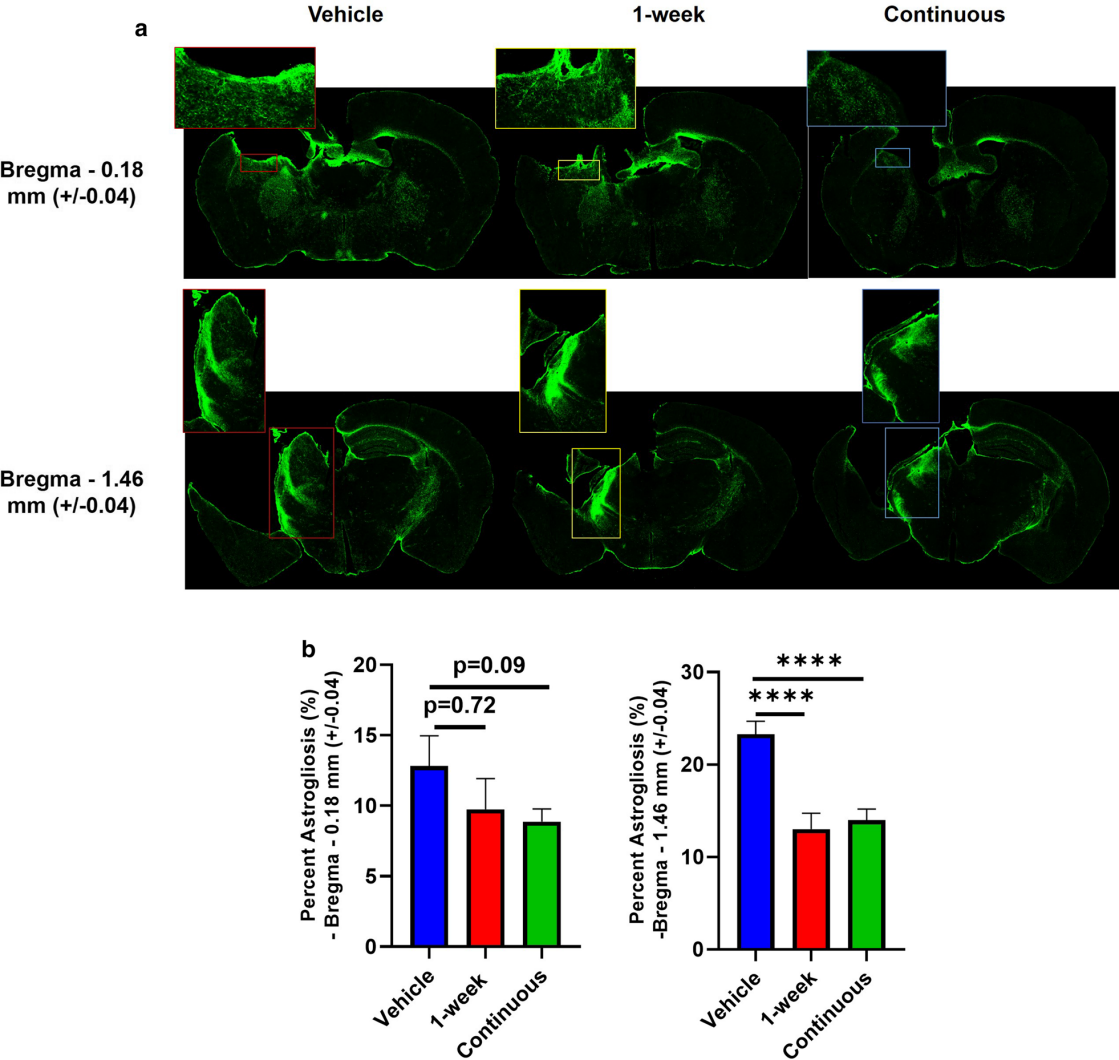
CR2Crry treatment decreases area of astrocytosis in the ipsilateral hemisphere. **a** 4x stitched immunofluorescence images of GFAP (green) staining in the three conditions of the study: vehicle, 1-week CR2Crry, and continuous CR2Crry from two different brain coordinates: Bregma − 0.18 mm (+/-0.04) and Bregma − 1.46 mm (+/-0.04). **b** Quantification of stained images. One-way ANOVA with Bonferroni correction for multiple comparisons. Unpaired T-test: continuous vs. vehicle. Error bars = Mean +/- SEM. ****p < 0.0001. n = 9–11/group.

A similar approach was used for analysis of astrogliosis using sections stained for GFAP (Fig. 5a). Compared to vehicle controls, the area of astrocytosis was significantly reduced in brains from mice continuously treated with CR2Crry at the Bregma − 1.46 mm (± 0.04) stereotactic location, and there was a strong trend in reduction at the − 0.18 mm (± 0.04) location (*p* value = 0.09). In brains from the 1 week treatment group, the difference only reached significance at the Bregma − 1.46 mm (± 0.04) location (Fig. 5b).

### Complement inhibition suppresses astrocyte recruitment and activation in the contralateral hippocampus of injured tissue

**Fig 6.**
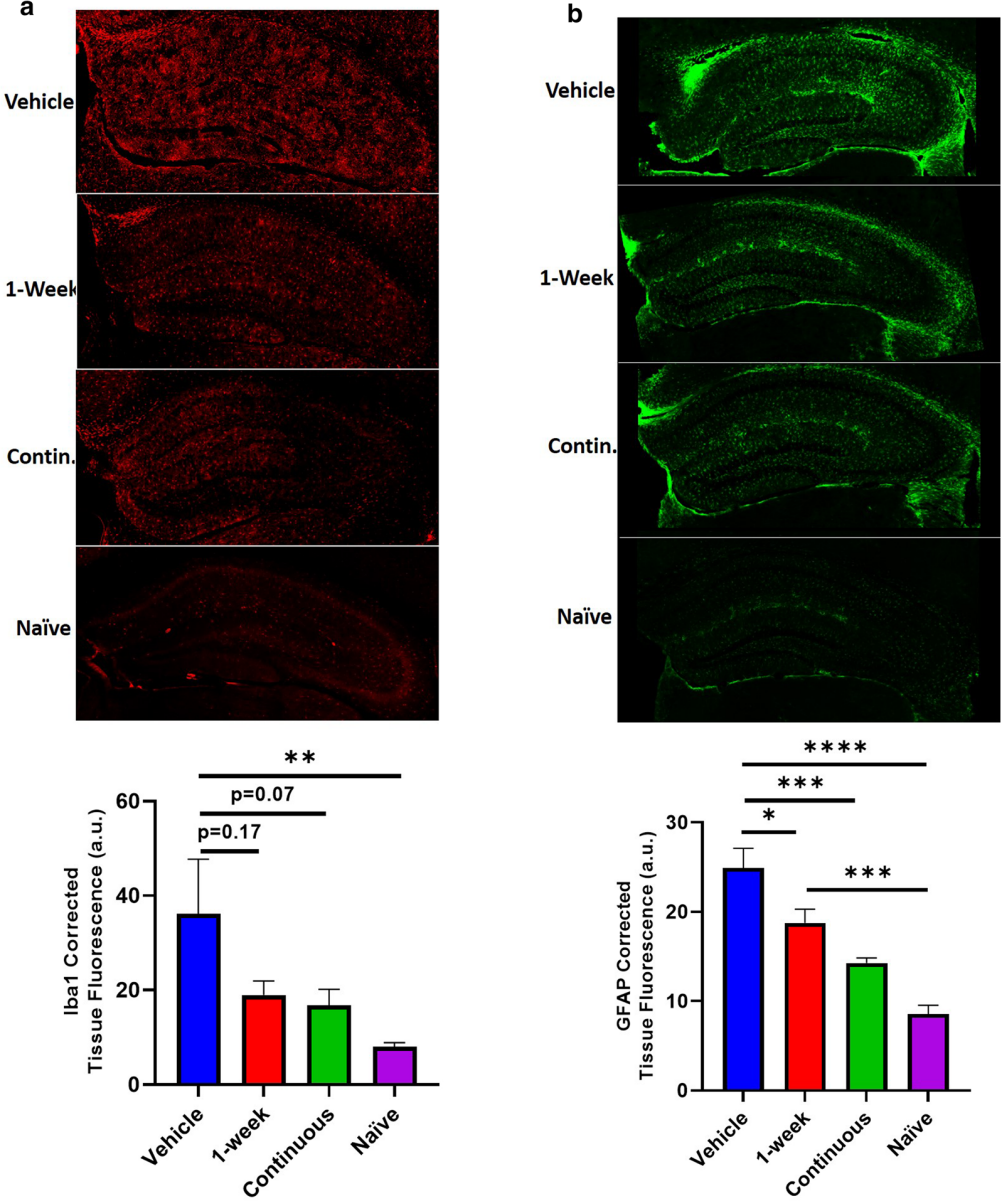
CR2Crry treatment decreases astrocytosis in the contralateral hippocampus. **a** Representative images of contralateral hippocampus Iba-1 staining for each of the following conditions: vehicle, 1-week CR2Crry, continuous CR2Crry, and naïve 9 month old mice. Quantification of Iba-1 intensity (corrected tissue fluorescence) is also shown. One-way ANOVA with Bonferroni correction for multiple comparisons. Unpaired T-test: continuous versus vehicle. Error bars = Mean +/- SEM. **p < 0.01. Vehicle, 1-week, continuous (n = 6–8) and naïve (n = 3). **b** Representative images of contralateral hippocampus GFAP staining for each of the following conditions: vehicle, 1-week CR2Crry, continuous CR2Crry, and naïve 9 month old mice. Quantification of GFAP intensity (corrected tissue fluorescence) is also shown. One-way ANOVA with Bonferroni correction for multiple comparisons. Unpaired T-test: continuous vs. vehicle. Error bars = Mean +/- SEM. *p < 0.05, ***p < 0.001, ****p < 0.0001. Vehicle, 1-week, continuous (n = 6–8) and naïve (n = 3).

In all groups studied, the CCI insult resulted in significant damage to the hippocampus, which plays an important role in cognition. We therefore investigated whether the involvement of the hippocampus in the contralateral hemisphere, which remains structurally intact, may contribute to the observed differences in Barnes maze performance in the different treatment groups. To this end, we analyzed the extent of microgliosis and astrocytosis in the contralateral hippocampus of all treatment groups. At 6 months after TBI, both microgliosis and astrocytosis was significantly increased in the contralateral hippocampus in brains from vehicle treated vs. naïve mice (Fig. 6a, b). Microgliosis, as measured by Iba1 staining intensity, showed a strong trend toward a decrease in the continuously treated group compared to vehicle, but did not reach significance (*p* value = 0.07). On the other hand, there was a significant decrease in astrocytosis in the contralateral hippocampus from both 1-week and continuous treatment groups. Taken together, these data reveal a potential link between hippocampal microgliosis and astrocytosis and decreased cognitive performance observed in vehicle treated animals, an outcome that can be reversed by complement inhibition.

## Discussion

There is strong evidence linking traumatic brain injury to cognitive decline and early onset dementia [7, 8]. The only currently practiced intervention is rehabilitation therapy, and there is no pharmacological treatment available. We recently demonstrated that transient (1-week) inhibition of complement (3 doses over 1 week) at 2 months after TBI interrupted a degenerative neuroinflammatory response and reversed cognitive decline measured at 3 months after TBI [2]. Here, we extend these studies to show that 6 months after TBI there is an ongoing expansion of complement-mediated neuroinflammation in the ipsilateral and contralateral brain, accompanied by cognitive decline. The data indicate that complement activation signals persist chronically after TBI and can reactivate a neuroinflammatory response subsequent to transient inhibition leading to ongoing cognitive decline. Therefore, this report documents via experimental manipulation that TBI should be recognized as a chronic pathology rather than a sequel of an acute insult, a finding that should guide further clinical translation of neuroprotective strategies that are to date limited to acute or transient treatments.

Transient administration of CR2Crry did not reverse cognitive decline at 6 months post TBI, in contrast to continuous periodic administration from 2 months through 6 months. On the other hand, continuous CR2Crry treatment did not improve motor performance compared to vehicle control, which is in line our previous data showing that locomotor function was similar in vehicle and CR2Crry treated mice at 3 months after injury [2]. It is possible that inclusion of additional approaches may be able to improve motor function, such as forced rehabilitation (e.g. powered treadmill or rotarod). A surprising result in our study was that in the open field ambulation test, vehicle animals travelled a longer distance than naïve animals. CR2Crry treated animals also showed a trend toward increased distance travelled. The open field test is used to measure both locomotor function and anxiety-like behavior in animals [43], and the increased distance travelled by injured mice is likely a reflection of a hyperactive state that is in line with previous studies in humans showing that TBI in student athletes is associated with attention deficit hyperactivity disorder (ADHD) [9]. Hyperactivity has been reported previously in murine TBI studies [39]. In a closed head injury model, increased locomotor hyperactivity was diminished upon acute treatment with minocycline, which correlated with reduced microglial activation [25]. Our data showed that complement inhibition decreased microglial activation together with a trend towards decreased distance on the open field task. The concept of potentially using a combination of therapeutics after TBI to reduce hyperactivity is an area for future work. In the context of experimental models, it would be interesting to investigate whether anxiety levels could be reduced by providing different configurations of the enriched environment or additives/alternatives to that which was utilized in the current study, such as bedding material changes and the providing of cardboard nesting boxes [22].

At 6 months post-TBI, lesion volume in the brains of mice continuously treated with CR2Crry starting 2 months after TBI was reduced compared to lesion volume in vehicle treated mice. This was not the case in brains from mice treated for 1-week with CR2Crry, and was not the case in our previous study in which brains were analyzed at 3 months after the same injury and treatment paradigm [2]. Of note, other studies in both a mouse model and rat model of CCI have shown an increase in lesion volume measured over 1 year [17, 31], and our data indicate that when administered chronically after TBI, ongoing complement inhibition is required to prevent lesion expansion. The CCI model we utilize in this study produces a large lesion, although large lesions after human TBI are not uncommon. In fact, human studies have shown that relatively large lesions at the time of insult are more likely to progress and grow [27]. In one study, 112 out of 491 patients that suffered from a TBI had a large infarct [14], and another study showed that 44 patients out of a total of 98 had a significant progression of lesion size as determined by CT scan [1]. Regardless, CR2Crry was able to significantly decrease a relatively large lesion in the murine TBI model used in this study. Lesion volume correlated with cognitive performance in that compared to vehicle, continuous CR2Crry treatment reduced lesion volume and improved cognitive performance in spatial learning and memory retention test, but 1-week CR2Crry treatment did neither.

In addition to lesion volume, improved cognitive performance in continuously treated mice also correlated with a reduced neuroinflammatory response, which expanded to both hemispheres by 6 months post-TBI. One measure of neuroinflammation was complement activation and C3 deposition in perilesional tissue, which was significantly reduced by continuous CR2Crry treatment, but not by 1-week treatment. The reduction in complement activation seen in the continuously treated group correlated with reduced microgliosis and astrocytosis in the ipsilateral hemisphere, and reduced astrocytosis in the contralateral hemisphere. In agreement with our finding of ongoing microglia activation at 6 months after injury, a previous study reported chronic microglial activation up to 1 year after TBI [31]. Also, depletion of microglia using a CSF1R inhibitor resulted in improved motor and cognitive performance at 3 months after TBI [24]. Notably, the CSFR1 inhibitor did not alter astrocytosis, which was evident in the cortex at 3 months after TBI. We have previously shown that microglia play a role in phagocytosis and elimination of C3 opsonized neurons acutely after stroke, and of C3 opsonized synapses after TBI, which is associated with cognitive decline [2, 3]. Others have also shown accumulation of C1q, which can initiate C3 activation and deposition, on synapses of aged mice after TBI was linked to microglial engulfment [29]. In the current study, we demonstrated that C3-NeuN and C3-NeuN-Iba1 interactions (colocalization) in the perilesional space were significantly reduced in the continuous, but not 1-week CR2Crry treatment groups. These findings correlated with improved cognitive function in continuous, but not 1-week treatment groups. The data is thus consistent with ongoing microglial phagocytosis of C3 opsonized neurons at 6 months after TBI, as has been reported at earlier time points after injury in stroke and TBI models.

Microglia are not the only cells implicated in phagocytosis and linked to cognitive decline. Astrocytes have also been shown to be key players in cognitive impairment in diseases such as Alzheimer’s disease [42] and experimental autoimmune encephalomyelitis [23]. The current data suggest that astrocytes may contribute to the chronic neuroinflammatory response after TBI, and which is in turn modulated by complement. This is an area for future investigation.

## Conclusions

In conclusion, there is a complement mediated expansion of lesion and neuroinflammatory response in the brain at 6 months after murine TBI, and is associated with cognitive deficit. Complement inhibition starting in the chronic phase (2 months) after TBI is effective at reducing neuroinflammation, reducing lesion size, and reversing cognitive decline when measured at 6 months post-TBI, but only if complement inhibition is sustained. Using a clinically relevant scenario of complement inhibition, the data indicate a role for both microglia and astrocytes in long-term cognitive decline after TBI, which is modulated by complement. The data strengthen the conclusion from a previous study that indicated complement inhibition in the chronic phase after TBI has potential as an effective therapeutic intervention, and has additional implications for potential translation in terms of patient management and treatment.

## Supplementary Information

**Additional file 1: Figiure S1.** a) Atlas image of with injury location. b) Photographs of an injured brain at day 180 along with a naïve age-matched brain

**Additional file 2: Figiure S2.** Atlas position (sagittal view) with selected regions used in staining and analysis throughout the manuscript drawn on the atlas position.

**Additional file 3: Figiure S1.** Bar graph comparing the learning slope (Path length vs. Time) of Vehicle, 1-Week, Continuous, and Naïve groups computed from Barnes Maze performance.

**Additional file 4: Video.** Rotating video depicting interactions between C3 (red), NeuN (green), and Iba1 (aqua).

## Data Availability

The datasets used and/or analyzed during the current study are available from the corresponding author on reasonable request.

## Abbreviations

C3: Complement component 3
CCI: Controlled cortical impact
EE: Enriched environment
GFAP: Glial fibrillary acidic protein
IACUC: Institutional Animal Care and Use Committee
Iba-1: Ionized calcium binding adaptor molecule 1
IP: Intraperitoneal
MAC: Membrane attack complex
NIH: National Institute of Health
PBS: Phosphate-buffered saline
PBST: Phosphate-buffered saline with 0.1% Triton-X
TBI: Traumatic brain injury
