# Supplementary figures and images for: Complement mediates neuroinflammation and cognitive decline at extended chronic time points after traumatic brain injury
Source: Acta Neuropathol Commun. 2021 Apr 20;9:72. doi: 10.1186/s40478-021-01179-6 (PMC8056513; doi:10.1186/s40478-021-01179-6)

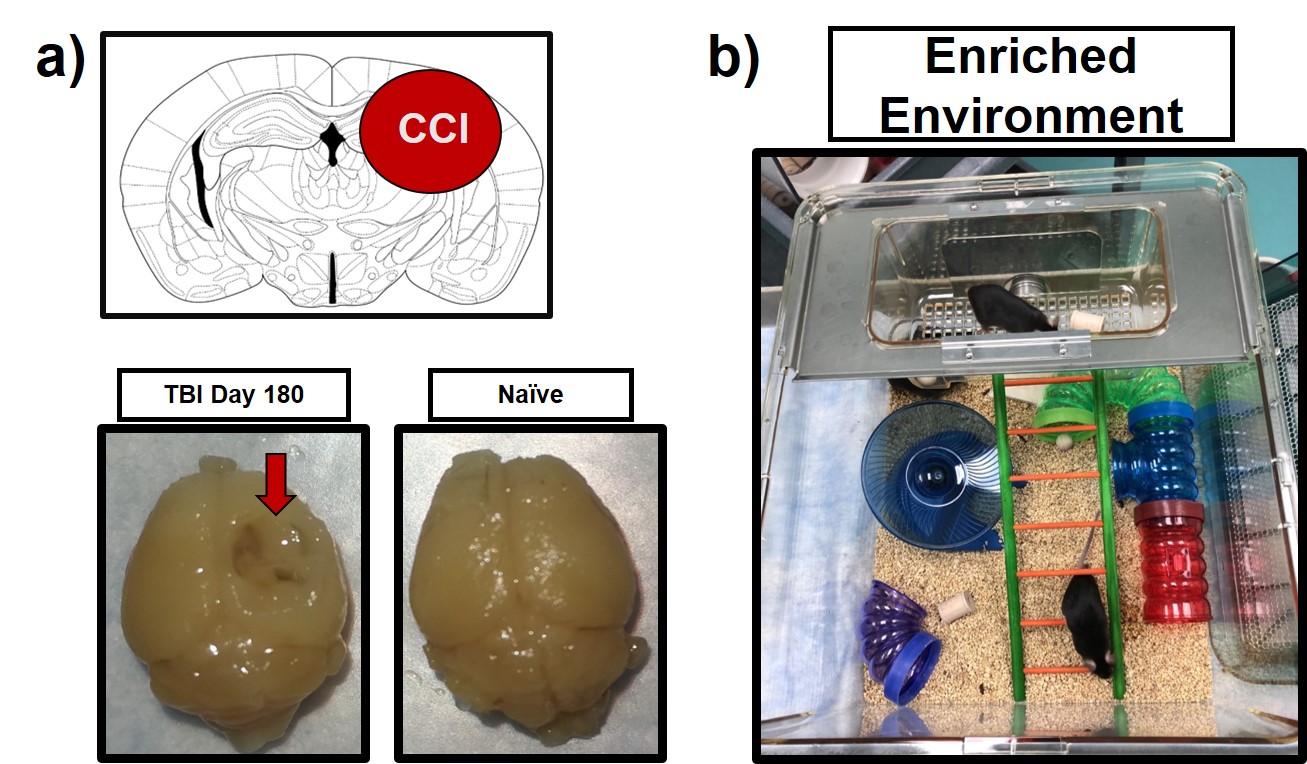

Supplement: Supplementary file 1 — Additional file 1: Figiure S1. a) Atlas image of with injury location. b) Photographs of an injured brain at day 180 along with a naïve age-matched brain [file 40478_2021_1179_MOESM1_ESM.jpg]

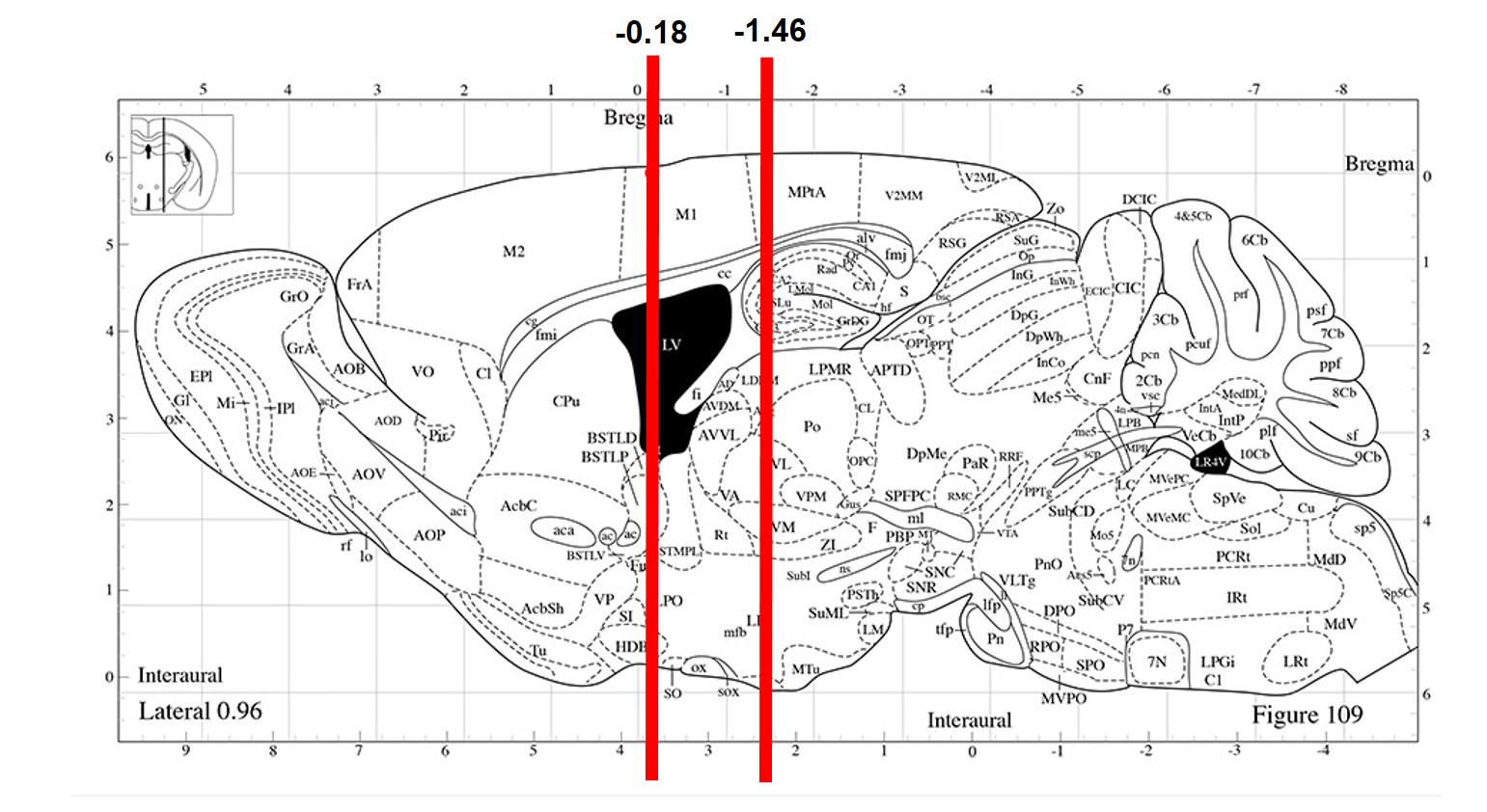

Supplement: Supplementary file 2 — Additional file 2: Figiure S2. Atlas position (sagittal view) with selected regions used in staining and analysis throughout the manuscript drawn on the atlas position. [file 40478_2021_1179_MOESM2_ESM.jpg]

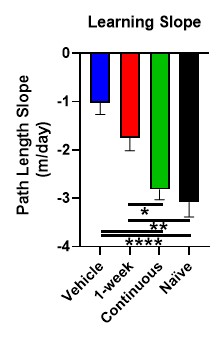

Supplement: Supplementary file 3 — Additional file 3: Figiure S1. Bar graph comparing the learning slope (Path length vs. Time) of Vehicle, 1-Week, Continuous, and Naïve groups computed from Barnes Maze performance. [file 40478_2021_1179_MOESM3_ESM.jpg]
